# Atlanta Academy of Medicine

**Published:** 1879-07

**Authors:** 


### ATLANTA ACADEMY OF MEDICINE.

ATLANTA, GA., May 25th, 1879.

Dr. Miller reported several cases of the ice cream poisoning. Said the symptoms were essentially those of ordinary cholera morbus, some mild, others severe, and attended by much and painful cramp in the extremeties. He called attention to the cases and gave his views of the cause of the attack. That there was some reason why so many persons, eating from the same place, at the same time, and should be similarly affected, admitted of no doubt. He had known a like train of symptoms follow in a number of persons, who partook of chicken salad or boiled custard. The symptoms in the cases following the ingestion of either the three articles of food—ice cream, boiled custard and chicken salad—were modified only by personal idiosincrasy identically the same.

The general idea that some metalic substance—lead, zinc, or copper—was the exciting cause, he repudiated in toto. There must be some one thing used in the preparation of all the articles to produce such like effects. In the preparation of chicken salad, no utensil or substance containing either lead, sinc, or copper was used, but instead an iron pot. The flavoring has been picked out by some as the mischievous agent, in the ice cream cases. The ordinary extracts of lemon, banana, vanilla, strawberry, etc., were used in giving flavor to the ice cream, but not to the chicken salad. Milk was used in the custard and cream, but not in the salad. Bacteria, or vegetable or animal germs of any kind, which might escape the boiling in the custard or salad, would in the ice cream be subjected to a degree of cold which would render them nil. Grant their power of surviving the caloric, this element of strength became one of weakness, when cold was applied.

There is one and only one common ingredient in the make up of the three substances under consideration, and that is eggs. Some people cannot eat eggs at all in any way, without being made sick by them, but to the large majority of people they are most wholesome, and easy of digestion when sound and fresh; but on the other hand a direct poison when unsound. The experiments of Matterchu had demonstrated that the process of endosmosis and exosmosis was immediately stopped by a very minute quantity of sulphuretted hydrogen gas, the very compound we had in the spoiled eggs. The first symptom generally in an ordinary case of cholera morbus, was the rotten egg belch. In private families poisoning from either of the articles of food under consideration was exceedingly rare, because more caution was used to reject the unsound eggs. In conversation with the owner of one of the saloons, where a number of persons were made sick from eating his cream, Dr. Miller asked him, what eggs he rejected in preparing the beverage. The reply was, all that smelt badly, or even those where the white and yellow of the egg had run together. Now we all know that the process of decomposition, of which the generation of sulphuretted hydrogen gas, is a process, and in the nascent state it is probably more deletorious than when once formed, has advanced considerably before the white and yellow commingle. True the process is arrested by the freezing operation, but just so soon as a high temperature is reached again—which is found in the stomach—the process is rapidly re-instituted more rapidly, because of the excess of heat in the stomach over that of the surrounding media. He took occasion to examine carefully the utensils in the same gentleman's saloon used to hold and make the cream. There were all of iron and block tin, save the ice box, which was lined with zinc.

Now as to treatment. The prognosis, if no medicine was given, was always favorable, indeed he was almost sure that to opium might be charged all fatal issues. Nature, by vomiting and purging, was doing her best to rid the system of the poison. So by all means assist her.

If after free and thorough evacuation of the entire alimentary canal, the cramps were troublesome, then and not until then would he venture to administer opium or alkaloids. He was satisfied that if dealers in ice cream, or proprietors of hotels knew the evil that lurked in an unsound egg, or one the "*least bit off*," for their own reputation, care in rejecting unsound eggs would be used, and we would hear of none or very few recurrences of these distressing cases. He was also sure of a favorble termination if we helped nature, and withheld opium until thorough evacuation of the deleterious substance.

---

Atlanta, May 26, 1879.

